# Development of Immunoassays for *Burkholderia pseudomallei* Typical and Atypical Lipopolysaccharide Strain Typing

**DOI:** 10.4269/ajtmh.16-0308

**Published:** 2017-02-08

**Authors:** Teerapat Nualnoi, Michael H. Norris, Apichai Tuanyok, Paul J. Brett, Mary N. Burtnick, Paul S. Keim, Erik W. Settles, Christopher J. Allender, David P. AuCoin

**Affiliations:** 1 Department of Microbiology and Immunology, University of Nevada School of Medicine, Reno, Nevada.; 2 Department of Infectious Diseases and Pathology, University of Florida, Gainesville, Florida.; 3 Department of Microbiology and Immunology, University of South Alabama, Mobile, Alabama.; 4 Department of Biological Sciences, Northern Arizona University, Flagstaff, Arizona.

## Abstract

*Burkholderia pseudomallei* is the causative agent of melioidosis, a severe infection endemic to many tropical regions. Lipopolysaccharide (LPS) is recognized as an important virulence factor used by *B. pseudomallei*. Isolates of *B. pseudomallei* have been shown to express one of four different types of LPS (typical LPS, atypical LPS types B and B2, and rough LPS) and in vitro studies have demonstrated that LPS types may impact disease severity. The association between LPS types and clinical manifestations, however, is still unknown, in part because an effective method for LPS type identification is not available. Thus, we developed antigen capture immunoassays capable of distinguishing between the LPS types. Mice were injected with B or B2 LPS for atypical LPS–specific monoclonal antibody (mAb) isolation; only two mAbs (3A2 and 5B4) were isolated from mice immunized with B2 LPS. Immunoblot analysis and surface plasmon resonance demonstrated that 3A2 and 5B4 are reactive with both B2 and B LPS where 3A2 was shown to possess higher affinity. Assays were then developed using capsular polysaccharide–specific mAb 4C4 for bacterial capture and 4C7 (previously shown to bind typical LPS) or 3A2 mAbs for typical or atypical LPS strain detection, respectively. The evaluations performed with 197 strains of *Burkholderia* and non-*Burkholderia* species showed that the assays are reactive to *B. pseudomallei* and *Burkholderia mallei* strains and have an accuracy of 98.8% (zero false positives and two false negatives) for LPS typing. The results suggest that the assays are effective and applicable for *B. pseudomallei* LPS typing.

## Introduction

*Burkholderia pseudomallei* is a Gram-negative saprophytic bacillus that is the causative agent of melioidosis, which is a life-threatening infectious disease prominent in southeast Asia and northern Australia.^1^,^2^ In the past decade, however, the number of melioidosis cases reported from other geographic locations such as India, China, and Brazil have increased, indicating that melioidosis is becoming a global problem.^2^–^5^ Due to its ability to cause a severe infection that may be transmitted by aerosol, *B. pseudomallei* has been recognized as a potential bioterrorism agent and has been classified as a Tier 1 select agent by the Centers for Disease Control and Prevention.^6^,^7^ Infection with *B. pseudomallei* results in high mortality rates that can be as high as 45%, even when medical interventions are provided.^8^ In addition, without appropriate antibiotic administration, the mortality rate could be as high as 90%.^9^ The absence of a licensed vaccine for prevention of melioidosis further impedes public health success.^10^

Lipopolysaccharide (LPS), a major outer membrane component of Gram-negative bacteria, is one of the most important virulence factors of *B. pseudomallei*.^11^ *Burkholderia pseudomallei* LPS is required for serum resistance; mutation in LPS biosynthetic genes can markedly attenuate the pathogen.^12^ Previous studies demonstrated that antibodies against *B. pseudomallei* LPS provide passive protection against melioidosis, whereas LPS–vaccinated mice survived lethal challenge, indicating that LPS is a protective antigen.^13^,^14^ As a result, development of a vaccine from this polysaccharide is an active focus in melioidosis research.^15^–^17^ The use of LPS as a vaccine target could be complicated by *B. pseudomallei* LPS structure diversity.

Structurally, LPS consists of lipid A, core oligosaccharide, and repeating units of immunogenic O-antigen. Based on seroreactivity, or an antibody response to LPS O-antigen, *B. pseudomallei* strains can be classified into two serotypes: 1) typical strains (producing typical or type A LPS), and 2) atypical strains (expressing atypical LPS, known as types B and B2), and a rough type (no serotype due to lack of O-antigen).^18^,^19^ LPS type B2 has been classified as an atypical type because of its cross-reactivity with serotype B patient sera; however, it expresses a ladder-banding pattern distinct from type B LPS.^19^ Thus, all four different types (type A, type B, type B2, and rough type) of *B. pseudomallei* strains possess unique LPS banding patterns that can be differentiated by sodium dodecyl sulfate polyacrylamide gel electrophoresis (SDS-PAGE).

Previous studies reported that *B. pseudomallei* strains producing different LPS types are epidemiologically different.^20^,^21^ The majority of *B. pseudomallei* strains are of the typical LPS type; however, 14.7% and 2.3% of strains isolated from northern Australia and southeast Asia, respectively, are of the atypical type (B, B2, or rough type).^19^ Distribution of *B. pseudomallei* strains in newly identified endemic areas such as the Indian subcontinent, southern China, Hong Kong, and Taiwan is still largely unknown.^4^,^5^,^22^ In addition, *B. pseudomallei* strains expressing different LPS types are believed to impact disease severity.^19^,^23^ Typical LPS has been found to be a weaker macrophage inducer compared with atypical LPS (G. Stephanie, unpublished data), potentially impacting disease prognosis and highlighting the need to distinguish the different LPS types of *B. pseudomallei* strains. Improved characterization would advance insight into the epidemiology and pathogenicity of *B. pseudomallei*, as well as guide melioidosis vaccine development. The aim of this study was to assist in these goals by developing a method that could efficiently differentiate between *B. pseudomallei* strains expressing different LPS types.

## Materials and Methods

### Bacterial cultures and preparation.

*Burkholderia pseudomallei* (174 strains), *B. pseudomallei* near-neighbor species (seven strains), *Acinetobacter baumannii* (15 strains), and *Pseudomonas aeruginosa* (one strain) were grown on Luria–Bertani (LB) agar plates at 37°C for 48 hours in a biosafety level (BSL)-3 facility (or a BSL-2 facility when appropriate). Colonies were picked and resuspended in 500 μL of phosphate-buffered saline (PBS) in O-ring gasketed microcentrifuge tubes. Bacterial samples then were inactivated by heating at 110°C for 15 minutes in a heat block. After heat inactivation, 50-μL samples were plated on LB agar plates and incubated for 48 hours to ensure sterility. After the sterility was confirmed, the samples were removed from the BSL-3 facility and refrigerated.

### Purification of LPS.

Atypical LPS types B and B2 were extracted from *Burkholderia ubonensis* strain MSMB57 and *Burkholderia thailandensis* strain 82172, respectively.^23^ Purification of LPS was performed as previously described using a kit-based method (Intron Biotechnology, Gyeonggi-do, Korea).^24^ Briefly, bacterial cells from an overnight culture on LB agar plates at 37°C were harvested and lysed in 20 mL of lysis buffer. The cells were vortexed to dissolve any cell clumps. After the addition of 4 mL chloroform, the sample was briefly vortexed and centrifuged for 1 hour at 4°C at 4,000 × *g*. The top aqueous layer was transferred to a clean tube and one volume each of LPS purification buffer and isopropanol were added to the aqueous layer. The sample was incubated overnight at −20°C prior to centrifugation at 4,000 × *g* for 1 hour at 4°C. The supernatant was removed and the pellet was resuspended in 5 mL of deionized water. The sample was dialyzed in water before being lyophilized. The lyophilized sample was stored at room temperature or suspended to 10 mg/mL in sterile water, and then stored at 4°C until use. LPS samples were treated with 50 μg/mL of DNase and RNase overnight at 37°C. The samples were further treated with 50 μg/mL proteinase K for 6 hours at 55°C to remove contaminating proteins. Treated samples were dialyzed in water overnight before lyophilization. The lyophilized LPS samples were sent to the University of Nevada, Reno for use in monoclonal antibody (mAb) production.

In addition to atypical LPS types B and B2, various types of LPS from *Burkholderia* spp. were used for mAb specificity determination. To obtain those LPS samples, culture media were inoculated with the *Burkholderia* strains listed in Table 1 (except *B. ubonensis* strain MSMB57 and *B. thailandensis* strain 82172) and incubated overnight at 37°C with vigorous shaking. Cell pellets were obtained by centrifugation and extracted using a modified hot aqueous-phenol procedure.^28^ Purified LPS antigens were then obtained essentially as previously described.^29^

### Immunization of mice and production of mAbs.

Atypical LPS-specific mAbs 3A2 (mouse IgG3) and 5B4 (mouse IgG1) were isolated from BALB/c mice immunized via subcutaneous injection with 50 μg purified B2 LPS with TiterMax Gold (TiterMax USA Inc., Norcross, GA) as an adjuvant. An indirect enzyme-linked immunosorbent assay (ELISA) was used to assess antibody titer to atypical LPS at weeks 4 and 6 postimmunization. Three days prior to harvesting of splenocytes, a final intravenous boost of 50 μg purified B2 LPS was administered. Hybridoma cell lines were generated using standard hybridoma techniques and were propagated in Integra CL 1000 culture flasks (Integra Biosciences, Hudson, NH).^30^ Hybridoma supernatant was collected and mAbs were purified using protein A affinity column chromatography. Isolation and production of typical LPS-specific mAb 4C7 (mouse IgG3) and capsular polysaccharide (CPS)–specific mAb 4C4 (mouse IgG1) have been described in our previous studies.^31^,^32^

### Immunoblot analysis.

Purified LPS samples (10 μg) were diluted in SDS-PAGE sample buffer and boiled for 10 minutes prior to electrophoresis on 12% TGX precast gels (Bio-Rad, Hercules, CA). Western blotting was performed with mini-nitrocellulose transfer packs and a Trans-Blot Turbo transfer system (Bio-Rad). The membranes were blocked with 5% skim milk in tris-buffered saline-Tween (TBS-T: 50 mM Tris, 150 mM NaCl, 0.1% Tween 20; pH 7.6) at 4°C overnight and incubated with 1 μg/mL of LPS-specific mAbs for 90 minutes at room temperature. After washing with TBS-T, the membranes were incubated with an anti-mouse IgG horseradish peroxidase (HRP) conjugate (Southern Biotech, Birmingham, AL) for 60 minutes at room temperature to facilitate detection. The final development was carried out using Pierce ECL Western Blotting Substrate (Pierce Biotechnology, Rockford, IL) and a ChemiDoc XRS imaging system (Bio-Rad).

### Indirect ELISA.

Microtiter plates were coated overnight with 100 μL of purified LPS (2 μg/mL in PBS) at room temperature. In cases where inactivated bacteria were used, the plates were coated overnight with killed bacterial suspensions diluted in PBS at room temperature. The plates were then washed with PBS-Tween (PBS containing 0.05% Tween 20) and blocked with a blocking solution (PBS containing 5% skim milk and 0.5% Tween 20) at 37°C for 1 hour. After blocking, the plates were washed with blocking solution, and then incubated with 100 μL of a 2-fold serial dilution of purified mAbs (or serum samples for antibody titer assessment) at room temperature for 90 minutes. After incubation, the plates were washed again with blocking solution, incubated with an anti-mouse IgG HRP conjugate at room temperature for 1 hour, followed by washing with PBS-Tween. The plates were developed by adding 100 μL of tetramethylbenzidine (TMB) substrate (KPL, Gaithersburg, MD) into each well. The reaction was stopped with 1 M H_3_PO_4_, and then the optical density at 450 nm (OD_450_) was read.

### Antigen capture immunoassay.

Antigen capture immunoassays to identify typical versus atypical LPS types for *B. pseudomallei* strains were developed. Microtiter plates were coated overnight at room temperature with 100 μL of mAb 4C4 (3 μg/mL in PBS), which is the mAb specific to *B. pseudomallei*/*Burkholderia mallei* manno-heptose CPS.^32^ The plates were washed with PBS-Tween and blocked with a blocking solution at 37°C for 1 hour. After blocking, the plates were washed with a blocking solution and 100 μL of killed bacterial samples were added to each well. The plates were then incubated at room temperature for 90 minutes, washed with PBS-Tween, and incubated at room temperature for 1 hour with 100 μL of 1 μg/mL of either the 4C7 HRP conjugate for typical strain (serotype A) detection or the 3A2 HRP conjugate for atypical strain (serotype B and B2) detection. After incubation, the plates were washed with PBS-Tween, developed with TMB substrate, and the reaction was stopped with 1 M H_3_PO_4_. The OD_450_ was read and the positive cutoff was set at 1.5, which was derived from the average OD_450_ plus three times the standard deviation of no cell controls. The assays were carried out in duplicate. The HRP-conjugated mAbs 4C7 and 3A2 used in these experiments were prepared using EZ-Link plus activated peroxidase kit (Pierce Biotechnology).

### Surface plasmon resonance.

Surface plasmon resonance (SPR) experiments were performed using a BIAcore X100 instrument (GE Healthcare, Piscataway, NJ) as previously described.^33^ Biotinylation of atypical LPS types B and B2 was carried out with EZ-Link Sulfo-NHS-LC-Biotin (Pierce Biotechnology). Biotinylated B and B2 LPS were separately immobilized onto streptavidin sensor chips (GE Healthcare). For each sensor chip, a flow cell was left unmodified for reference subtraction. The analysis was conducted by using 1× HBS-EP+ (10 mM N-[2-hydroxyethyl]piperazine-N′-[2-ethanesulfonic acid], 150 mM NaCl, 3 mM ethylenediaminetetraacetic acid, and 0.05% v/v Surfactant P20; GE Healthcare) as a running buffer and diluent. To evaluate binding affinity, at least six different concentrations of mAbs were used. For each running cycle, the mAb was injected over the surface of a sensor chip at a flow rate of 30 μL/minute for 180 seconds. After this time, the mAb was allowed to passively dissociate for 300 seconds. The sensor surface was regenerated between runs with a 30-second pulse of 10 mM NaOH to ensure the removal of residually bound mAb. The steady-state affinity (*K*_D_) was determined using a steady-state model in BIAevaluation software version 2.0.1 (GE Healthcare). Accuracy of the model fitting was described by χ^2^ parameter calculated by the BIAevaluation software.

## Results

### Specificity of mAbs.

**Figure 1.**
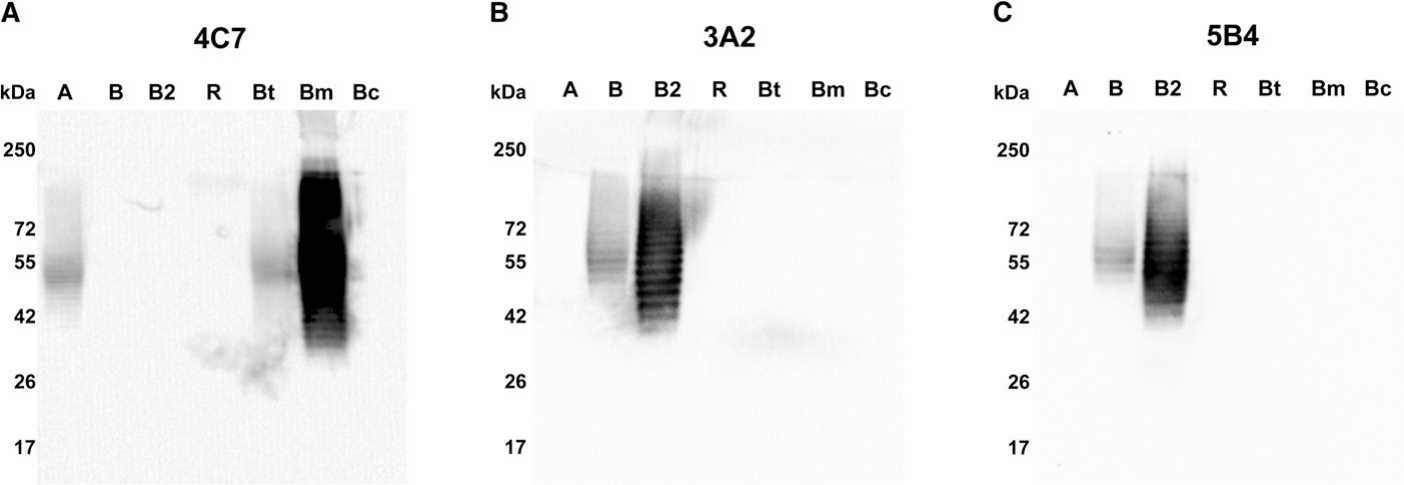
Immunoblot analysis to determine specificity of monoclonal antibodies (mAbs) for various lipopolysaccharide (LPS) types. Sodium dodecyl sulfate polyacrylamide gel electrophoresis (SDS-PAGE) gels were loaded with 10 μg of *Burkholderia pseudomallei* LPS type A (A), type B (B), type B2 (B2), rough type (R), *Burkholderia thailandensis* LPS (Bt), *Burkholderia mallei* LPS (Bm), and *Burkholderia cenocepacia* LPS (Bc). After blotting, the membranes were probed with mAbs 4C7 (**Panel A**), 3A2 (**Panel B**), or 5B4 (**Panel C**). MAb 4C7 reacted with type A LPS with no cross-reactivity to type B and B2 LPS. LPS from *B. thailandensis* E264 and *B. mallei,* which are known to have similar type A LPS, were also reactive with mAb 4C7. In contrast, mAbs 3A2 and 5B4 bound specifically to atypical *B. pseudomallei* LPS (both type B and B2) with no cross-reactivity to other LPS types.

Specificity of typical LPS mAb 4C7 and atypical LPS mAbs 3A2 and 5B4 was analyzed by immunoblot analysis for their target LPS type. Immunoblots of LPS from various strains of *Burkholderia* species probed with mAb 4C7 demonstrated that the antibody was reactive to typical *B. pseudomallei* LPS type A, with no cross-reactivity to atypical LPS type B or B2 (Figure 1A

). Reactivity of mAb 4C7 to LPS from *B. thailandensis* E264 was expected since it has been shown previously that *B. thailandensis* E264 expresses typical *B. pseudomallei* LPS.^23^,^26^ In addition, *B. mallei* LPS, which is also known to be structurally similar to *B. pseudomallei* LPS type A, was recognized by mAb 4C7 as anticipated.^23^,^34^ In contrast to mAb 4C7, mAbs 3A2 and 5B4 recognized atypical LPS types B and B2, and had no cross-reactivity to type A LPS (Figure 1B and C).

### Binding affinity of mAbs by SPR.

**Figure 2.**
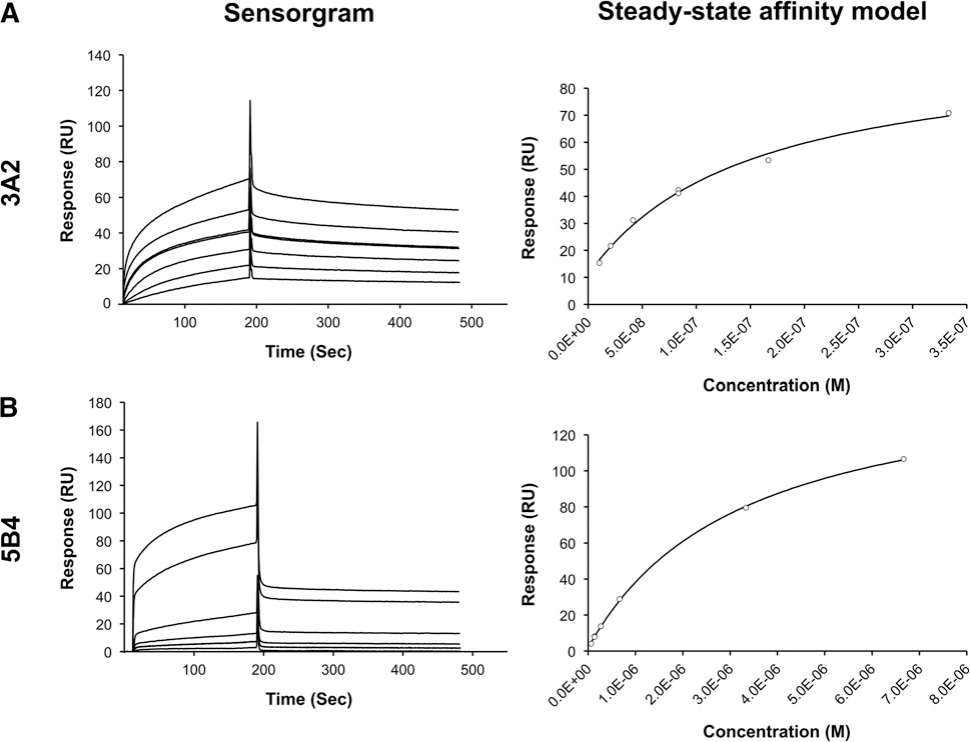
Surface plasmon resonance (SPR) analysis of binding affinity between monoclonal antibodies (mAbs) 3A2 and 5B4 to immobilized type B lipopolysaccharide (LPS). Biotinylated LPS type B was immobilized on the surface of streptavidin (SA)-coated sensor chip with the final response units of 306.2. The sensorgrams (left panel) were obtained by injecting the mAbs 3A2 (10–333 nM, **Panel A**) and 5B4 (67–6,667 nM, **Panel B**) over the chip surface for 180 seconds followed by passive dissociation for 300 seconds. Right panel presents steady-state affinity model fitting of each mAb.

**Figure 3.**
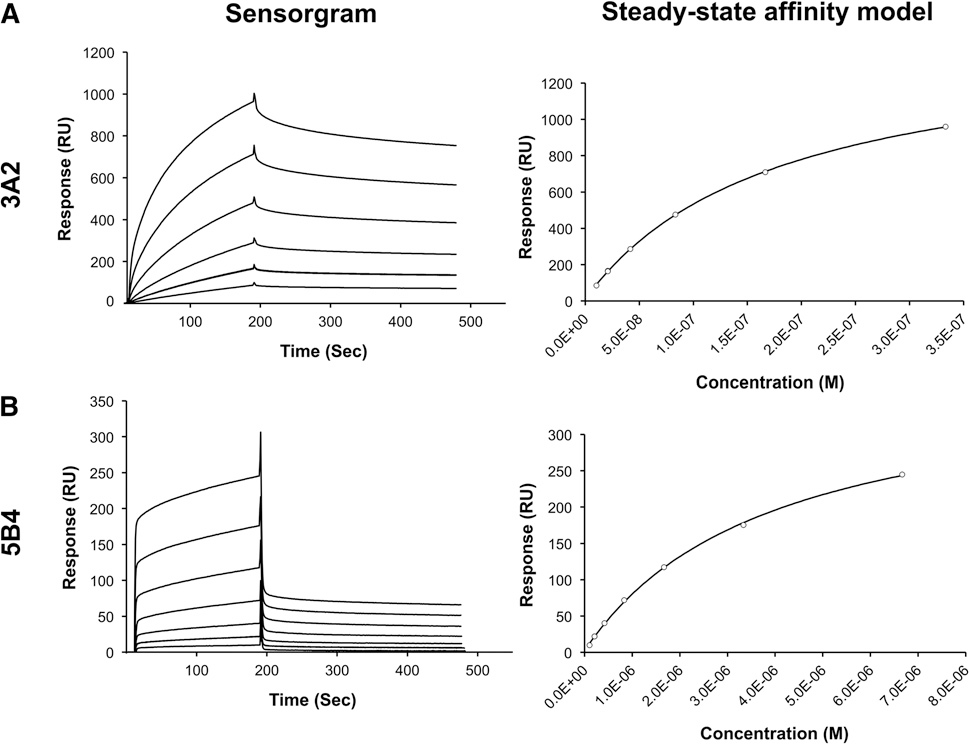
Surface plasmon resonance (SPR) analysis of binding affinity between monoclonal antibodies (mAbs) 3A2 and 5B4 and immobilized type B2 lipopolysaccharide (LPS). Biotinylated LPS type B2 was immobilized on the surface of streptavidin (SA) sensor chip with the final response units of 523.1. The sensorgrams (left panel) were obtained by injecting the mAbs 3A2 (10–330 nM, **Panel A**) and 5B4 (100–6,667 nM, **Panel B**) over the chip surface for 180 seconds followed by passive dissociation for 300 seconds. Right panel presents steady-state affinity model fitting of each mAb.

SPR was used to study binding affinity of mAbs 3A2 and 5B4 for LPS. Each mAb was analyzed at several concentrations over the surface of a type B LPS–coated sensor chip (Figure 2

) and a B2 LPS–coated sensor chip (Figure 3

) as described. The data from the SPR sensorgrams (left panels in Figures 2 and 3) were fitted to the nonlinear steady-state affinity model (right panels) to obtain the *K*_D_ values. Fitted models were considered to be accurate based on χ^2^ (< 10% of R_max_). The *K*_D_ values of mAbs 3A2 and 5B4 binding to immobilized B and B2 LPS are summarized in Table 2. The binding affinity of mAb 3A2 to B LPS (*K*_D_ = 149 nM) and B2 LPS (*K*_D_ = 177 nM) is comparable. Also, the affinity of mAb 5B4 binding to LPS type B (*K*_D_ = 3,262 nM) and B2 (*K*_D_ = 3,897 nM) is apparently similar. The results indicate that mAbs 3A2 and 5B4 have no binding preference between LPS type B and B2. However, mAb 3A2 shows a higher affinity binding to atypical LPS compared with mAb 5B4.

### Strain typing by antigen capture immunoassay.

**Figure 4.**
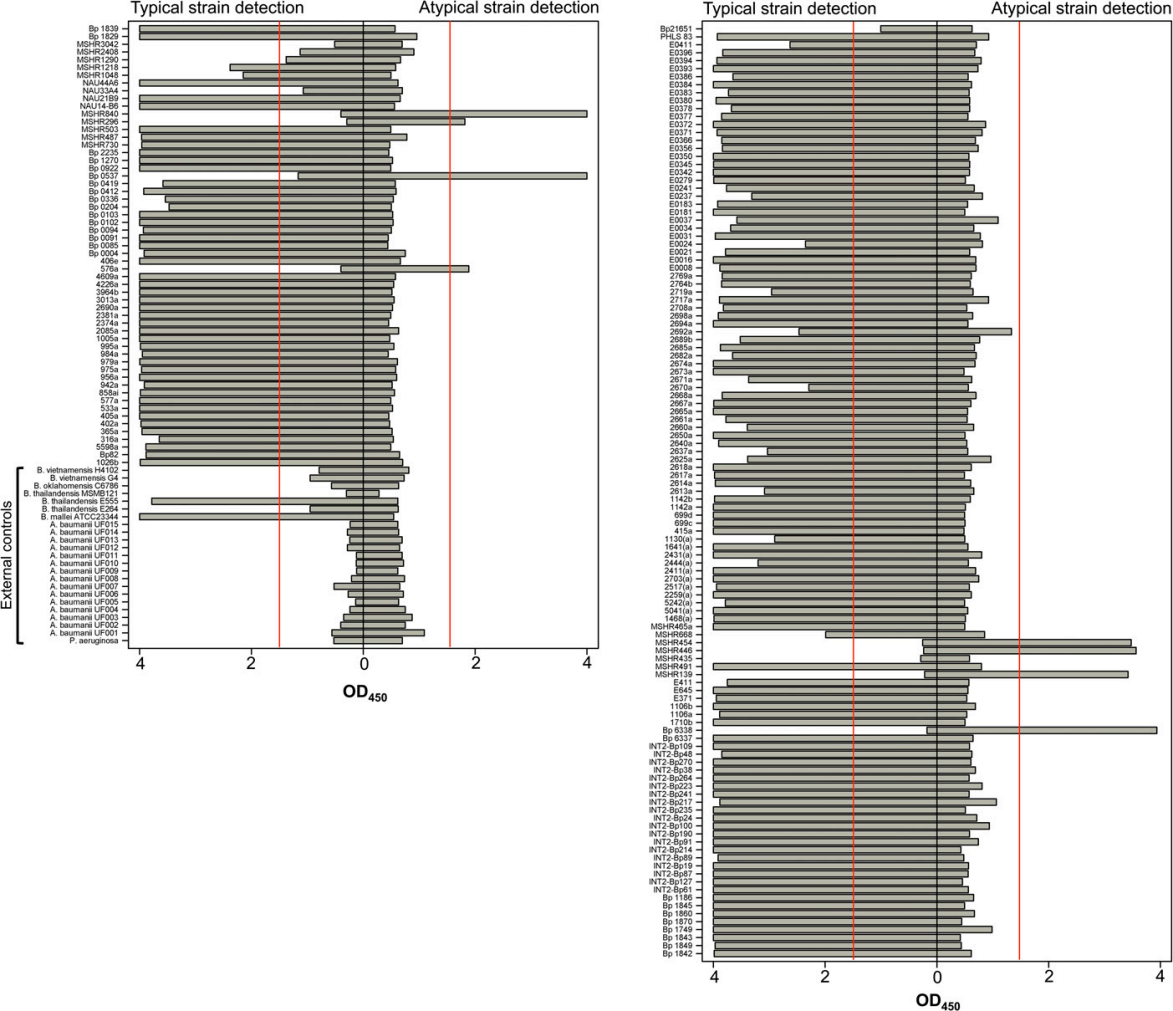
Detection of typical and atypical *Burkholderia pseudomallei* strains by antigen capture immunoassay. Whole killed bacteria were captured with capsular polysaccharide (CPS)–specific monoclonal antibody (mAb) 4C4 coated on microtiter plates. The strains were detected with either typical lipopolysaccharide (LPS)–specific mAb 4C7 horseradish peroxidase (HRP) conjugate (left) or atypical LPS–specific mAb 3A2 HRP conjugate (right). Strains that have an OD_450_ of 1.5 (red line) or greater were considered positive for the assay and designated as typical or atypical strains.

Two antigen capture immunoassays were developed for typical and atypical *B. pseudomallei* strain typing. To detect typical LPS (type A) strains of the bacteria, CPS-specific mAb 4C4 and typical LPS-specific mAb 4C7 HRP conjugate were used as capture and detector mAbs, respectively. An immunoassay to detect atypical LPS (type B or B2) *B. pseudomallei* strains was developed by replacing 4C7 HRP conjugate with atypical LPS-specific mAb 3A2 HRP conjugate (capture mAb 4C4 remained the same). The assays were evaluated with 197 strains of inactivated bacteria including clinical and environmental *B. pseudomallei*, other *Burkholderia* spp. (including close genetic relatives of *B. pseudomallei*), and clinical isolates of *P. aeruginosa* and *A. baumannii* from endemic areas used as external controls. The results for detection of typical and atypical *B. pseudomallei* strains are presented in Figure 4

**Figure 5.**
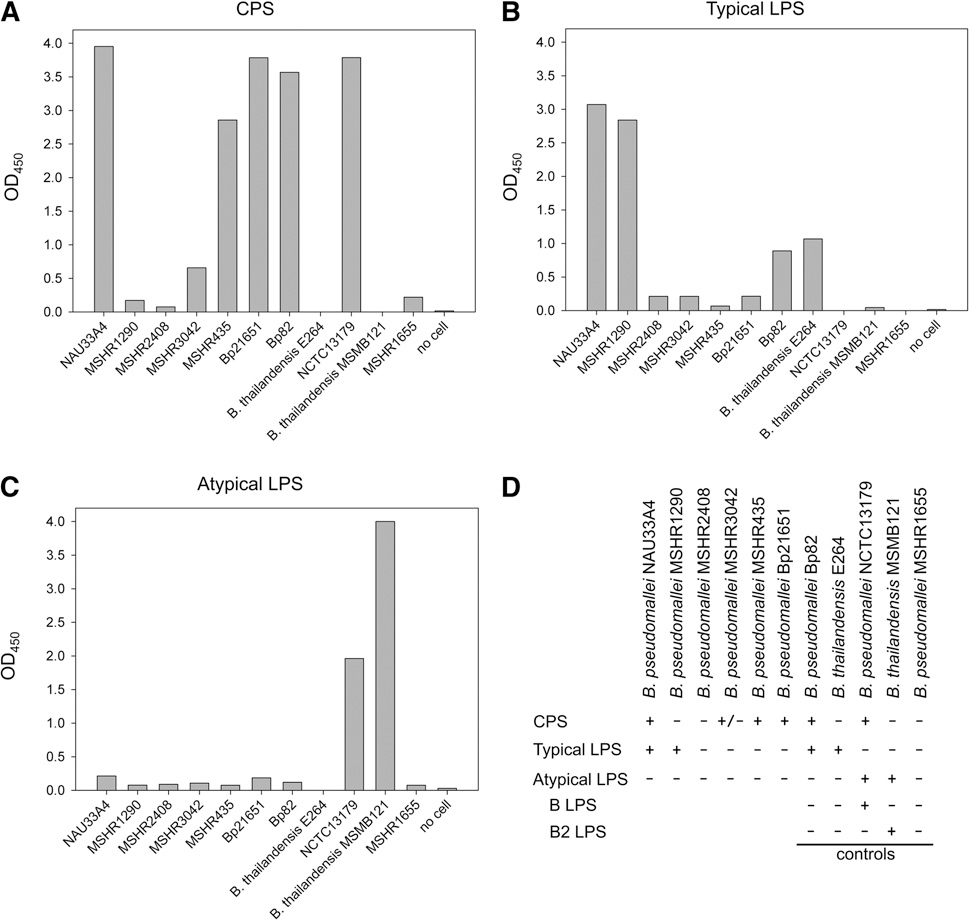
Phenotypic analysis of capsular polysaccharide (CPS) and lipopolysaccharide (LPS) by indirect enzyme-linked immunosorbent assay (ELISA). Double-negative *Burkholderia pseudomallei* strains in the capture ELISA were used to coat microtiter plates. The strains were detected with monoclonal antibodies (mAbs) 4C4 (**Panel A**), 4C7 (**Panel B**), and 3A2 (**Panel C**) for detection of CPS, typical LPS, and atypical LPS expression, respectively. **Panel D** presents the result summary. The results show that NAU33A4 and MSHR1290 are typical type, and MSHR2408, MSHR3042, MSHR435, and Bp21651 are rough type. Bp82, *Burkholderia thailandensis* E264, NCTC13179, MSHR1655, and *B. thailandensis* MSMB121 were used as controls.

. Strains that yielded the OD_450_ of 1.5 or greater were considered to be positive for the assays (Figure 4, Supplemental Table 1). Clinical isolates of *P. aeruginosa* and *A. baumannii*, non-*Burkholderia* spp. controls, were negative for both typical and atypical strain detection. The results also demonstrated that most of the strains that were negative for typical strain detection were positive in the atypical strain assay (Figure 4). None of the strains tested in this study were positive for both assays (double positive), suggesting that there is no occurrence of a false-positive result. However, six strains of *B. pseudomallei* were negative with both typical and atypical strain detection assays (double negative); thus, their strain types could not be identified by the immunoassays. Even though the double-negative result could be a false-negative error from the assays, it could also be a consequence of certain isolates lacking O-antigen (rough type LPS) and/or CPS in those strains. Thus, to investigate further whether the double-negative results were a result of false negatives, the strains that gave double-negative results were analyzed phenotypically using indirect ELISA (Figure 5

). The analysis revealed that strains MSHR2408, MSHR3042, MSHR435, and Bp21651 were rough type that did not produce O-antigen. Of 174 strains tested, only two typical strains (NAU33A4 and MSHR1048) were negative with typical strain detection. This is possibly because CPS expression levels (Figure 5) or numbers of bacterial cells in those samples were low. Nevertheless, we have considered these two strains as false-negative results.

## Discussion

It has been reported for many years that *B. pseudomallei* expresses various LPS structures.^28^ The relationship between *B. pseudomallei* strains producing different LPS types and their endemic areas has been documented, but is not yet complete.^19^ The association between LPS types and disease severity or clinical manifestations, however, has never been reported. This is possibly because current methods used for determining the various LPS types have many limitations. For example, SDS-PAGE with silver staining requires bacterial samples to be lysed and treated with proteinase K prior to electrophoresis.^18^ In addition, serological typing by immunoblot (a Western blot of bacterial samples probed with the serotype-specific melioidosis patient serum) is limited by availability of a patient's serum, whereas its sensitivity and accuracy rely on the amount and specificity of polyclonal antibody in the serum.^19^ Limitations of the current techniques led us to the development of more effective and reliable assays for *B. pseudomallei* strain typing.

In this study, we developed a set of antigen capture immunoassays for typical and atypical *B. pseudomallei* LPS detection utilizing mAbs specific to typical and atypical *B. pseudomallei* LPS. To obtain atypical LPS–specific mAbs, we immunized mice with purified LPS type B or B2. Two mAbs, 3A2 and 5B4, were isolated from mice immunized with B2 LPS. Unfortunately, we could not isolate any mAb from type B LPS-immunized mice. In a second attempt to isolate B LPS mAbs, we immunized mice with heat-inactivated *B. ubonensis* strain MSMB75, but we were still unable to isolate mAbs from these mice (data not shown). However, 3A2 and 5B4 mAbs isolated from B2 LPS-immunized mice do cross-react to type B LPS (Figure 1B and C). We further investigated the cross-reactivity of all sera from the immunized mice (Supplemental Figure 1). Some of the sera from B LPS-immunized mice were found to cross-react with B2 LPS, which was consistent with the cross-reactivity reported from serotype B patient sera.^19^,^21^ We also found that the sera from B2 LPS-immunized mice were reactive to B LPS, which corresponds to the cross-reactivity we have seen in 3A2 and 5B4 mAbs. Nevertheless, Western blot analysis described by Sorenson and others demonstrated that sera from patients infected with B2 LPS strains were not cross-reactive with B LPS.^21^ We also acknowledge that diversity in antibody responses is very common, even among individuals within the same species (Supplemental Figure 1).^35^ Thus, it is possible that humans and mice may recognize LPS epitopes distinct from each other. Altogether, these results suggest that the O-antigen structures of B and B2 LPS are very similar and contain common epitopes. Therefore, it is difficult to isolate mAbs capable of distinguishing between B and B2 LPS. To achieve this goal, information about structures and biosynthesis of LPS might be required.

We also investigated the cross-reactivity of mAbs 3A2 and 5B4 with typical LPS (type A), and found no cross-reactivity (Figure 1B and C). As expected, typical LPS-specific mAb 4C7 was not cross-reactive to atypical LPS as well (Figure 1A). These mAbs showed no cross-reactivity to *Burkholderia cenocepacia* LPS or the rough type LPS of *B. pseudomallei*, confirming that they are specific to typical or atypical LPS O-antigens. The reactivity of mAb 4C7 to LPS purified from *B. thailandensis* E264 and *B. mallei* was also observed (Figure 1A). This result was not surprising since *B. thailandensis* E264 is known to produce the same LPS structure as that produced by typical *B. pseudomallei*.^26^ In addition, the structures of typical *B. pseudomallei* and *B. mallei* LPS are very similar, except that *B. mallei* LPS lacks the 4-*O*-acetyl modifications on the 6-deoxy-α-L-talopyranose.^25^ We noted that mAb 4C7, which was isolated from inactivated *B. pseudomallei* 1026b–immunized mice,^31^ reacted to *B. mallei* LPS more strongly than typical *B. pseudomallei* LPS (Figure 1A) possibly because the absence of the 4-*O*-acetylation in the *B. mallei* LPS structure makes its epitopes more accessible to the mAb.

3A2 and 5B4 are two mAbs specific to atypical *B. pseudomallei* LPS. To decide which mAb should be incorporated into an atypical strain detection immunoassay, SPR was used. Steady-state affinity (*K*_D_) values derived from SPR demonstrated that 3A2 and 5B4 exhibit no binding selectivity between B and B2 LPS (Table 2). However, the binding affinity of mAb 3A2 to the both LPS antigens is roughly 20-fold greater than that of mAb 5B4. In addition, the sensorgrams showed the difference in the kinetics of binding between these two mAbs (Figures 2 and 3). According to the sensorgrams, 3A2 exhibited slower dissociation rates compared with 5B4, whereas the association rates were apparently comparable. This was consistent with the greater affinity of 3A2 demonstrated by the steady-state affinity model. Therefore, we selected mAb 3A2 to incorporate into the immunoassay.

In this study, the antigen capture immunoassays (sandwich ELISA) were developed using mAbs 4C7 and 3A2 for typical and atypical strain detection, respectively, whereas CPS-specific mAb 4C4 was used for bacterial capture. We chose to capture whole cell bacteria with the mAb because it is known to be highly specific to *B. pseudomallei*.^33^ CPS is another cell-surface component of many Gram-negative bacteria. The presence of CPS is closely related to the pathogenicity of the organism.^36^ mAb 4C4, recognizes a CPS epitope on within its structure of an unbranched homopolymer of 1,3-linked 2-*O*-acetyl-6-deoxy-β-D-*manno-*heptopyranose.^32^ There are five different CPS structures that are potentially produced by *B. pseudomallei*; however, this structure appears to be a potent virulence factor, along with being well conserved.^11^ The same CPS structure is also found in *B. mallei*.^37^ Thus, using CPS-specific mAb 4C4 to capture creates increased selectivity toward pathogenic *Burkholderia* strains. Since *B. mallei* produces a typical *B. pseudomallei*-like LPS, it is important to note that the typical strain detection immunoassay will not be able to differentiate between typical strains of *B. pseudomallei* and *B. mallei*.

A total of 197 strains of bacteria (as listed in Supplemental Table 1) were used to investigate the performance of the assays. We found that the clinical isolates of *P. aeruginosa* and *A. baumannii* were negative for both typical and atypical strain detection. In addition, all nonpathogenic *B. pseudomallei* near-neighbors (except *B. thailandensis* E555, which is known to produce *B. pseudomallei*-like CPS) were not detected by the assays.^38^ In contrast, nearly all *B. pseudomallei* strains tested were detected by either typical detection or atypical detection assay. Together, the results suggest that our immunoassays are highly specific to pathogenic *Burkholderia.* The results also suggest that the CPS antigen recognized by mAb 4C4 is highly conserved in *B. pseudomallei* species; these results correspond with our previous studies performed on a large bacterial panel using a different CPS-specific mAb.^33^ Among 174 *B. pseudomallei* strains tested, 63 of them have their LPS types published. We observed a near-perfect matching (62 of 63 strains) between the strain-typing results from our immunoassays and the published LPS types, indicating that the assays are highly accurate (Supplemental Table 1 and Table 3). The one strain that was mismatched is typical strain MSHR1290, and it was revealed later that the strain expressed a low level of CPS (Figure 5). From 111 strains of unknown LPS phenotypes, the immunoassays were able to clearly designate the strain type in 107 of them (Table 3). For the other four strains, the assays yielded double-negative results; thus, their LPS types could not be identified. However, three subsequent indirect ELISAs were able to reveal their CPS and LPS phenotypes (Figure 5). According to the indirect ELISA result, only NAU33A4 was considered as a false-negative error of the assays. Altogether, of 174 strains tested, we observed only two false negatives, and no false positives, yielding an accuracy of 98.8%. In this study, we have demonstrated that our immunoassay is effective and applicable for identifying different LPS types of *B. pseudomallei* strains. Use of these assays following an established method of *B. pseudomallei* identification such as latex agglutination or lateral flow immunoassay (LFI) would provide helpful information for clinicians involved in melioidosis research.^9^,^33^ Potentially, the antigen capture immunoassay could be adapted to the LFI format. This would provide rapid LPS typing and may provide clinicians important information if it is discovered that variable LPS types correspond to changes in virulence.

## Conclusions

To our knowledge, this is the first development of immunoassays (sandwich ELISAs) for *B. pseudomallei* typical versus atypical strain typing using mAbs specific to typical and atypical LPS. The immunoassays have demonstrated a high accuracy in identification of *B. pseudomallei* strain types. Compared with previous methods (silver-stained SDS-PAGE and serological typing immunoblot), the immunoassays require less sample preparation, and are more reliable as patient serum is no longer required. In addition, as in an ELISA platform, the new assays are more suitable for screening a large strain panel of bacteria. By using CPS-specific mAb in the capturing phase, we can detect nearly all strains of pathogenic *B. pseudomallei*. It is important to note that the assays could not differentiate between typical *B. pseudomallei* and *B. mallei*, but the ability of the assay to detect select agent *Burkholderia* spp. is not diminished. It is also important to emphasize that the assays developed in this study are not intended for use as a primary method of *B. pseudomallei* identification, since a negative reading from the assays could be interpreted as either *B. pseudomallei* expressing rough type LPS or other Gram-negative bacteria. Rather, the assays were designed for use following an established method of *B. pseudomallei* detection for identification of LPS type strains among different *B. pseudomallei* strains. Additionally, our immunoassays could not distinguish between rough type strains and CPS-negative strains, as both of them yielded negative results by both typical and atypical detection assays. However, those strains exist as a small proportion of the known *B. pseudomallei* population, and indirect ELISAs (or Western blotting) can be used to reveal their CPS/LPS phenotypes easily, as demonstrated in Figure 5. Overall, the antigen capture immunoassays are an efficient method for *B. pseudomallei* typical and atypical strain typing, which could advance epidemiological study as well as our understanding of pathogenesis in particular types of *B. pseudomallei* infection and facilitate LPS-based melioidosis vaccine research.

## Supplementary Material

Supplemental table and figure.

## Figures and Tables

**Table 1:** *Burkholderia* strains used for mAb specificity determination

Species	Strain	Description	Reference
*Burkholderia pseudomallei*	RR2808	Bp82 derivative; Δ*wcbB*; expresses Type A LPS	25
*B. pseudomallei*	RR5491	Bp82 derivative; Δ*wcbB*Δ*rmlD*; expresses rough LPS	P. J. Brett, unpublished
*Burkholderia thailandensis*	E264	Environmental isolate; expresses Type A LPS	26
*Burkholderia mallei*	BM2308	ATCC 23344 derivative; Δ*wcbB*	25
*Burkholderia ubonensis*	MSMB57	Expresses type B LPS	23
*B. thailandensis*	82172	Expresses type B2 LPS	23
*Burkholderia cenocepacia*	K56-2	CF sputum isolate	27

CF = cystic fibrosis; mAb = monoclonal antibody; LPS = lipopolysaccharide.

**Table 2:** Summary of SPR analysis results

Antigen	mAb 3A2			mAb 5B4		
	*K*_D_ (nM)	Rmax (RU)	χ^2^ (RU^2^)	*K*_D_ (nM)	Rmax (RU)	χ^2^ (RU^2^)
B-LPS	149	85	3.1	3,262	155	0.5
B2-LPS	177	1,453	16	3,897	382	4.6

LPS = lipopolysaccharide; *K*_D_ = steady-state affinity; mAb = monoclonal antibody; RU = response units; SPR = surface plasmon resonance.

**Table 3:** Summary of *Burkholderia pseudomallei* strains classified by LPS type and antigen capture immunoassay results

*B. pseudomallei* strains	Total	LPS types				Antigen capture immunoassay results			
		A	B	B2	Rough	Typical strains	Atypical strains	Double positive*	Double negative†
Known LPS types	63	57	1	4	1	56	5	0	2‡
Unknown LPS types	111					104	3	0	4§
Total	174					160	8	0	6

CPS = capsular polysaccharide; LPS = lipopolysaccharide.

* The number of strains that were positive by both typical and atypical strain detection assay, which could be a result of false-positive error of the assays.

† The number of strains that were negative by both typical and atypical strain detection assay, which could be a result either false-negative error of the assays or no CPS/LPS expression.

‡ Two strains: MSHR1290 (false negative) and MSHR435 (rough type).

§ Four strains: NAU33A4 (false negative), MSHR2408 (rough type), MSHR3042 (rough type), and Bp21651 (rough type).
