# Supplementary material for: Development of Immunoassays for Burkholderia pseudomallei Typical and Atypical Lipopolysaccharide Strain Typing
Source: Am J Trop Med Hyg. 2017 Feb 8;96(2):358–67. doi: 10.4269/ajtmh.16-0308 (PMC5303037; doi:10.4269/ajtmh.16-0308)
Supplement: Supplementary file 1 [file SD5.pdf]

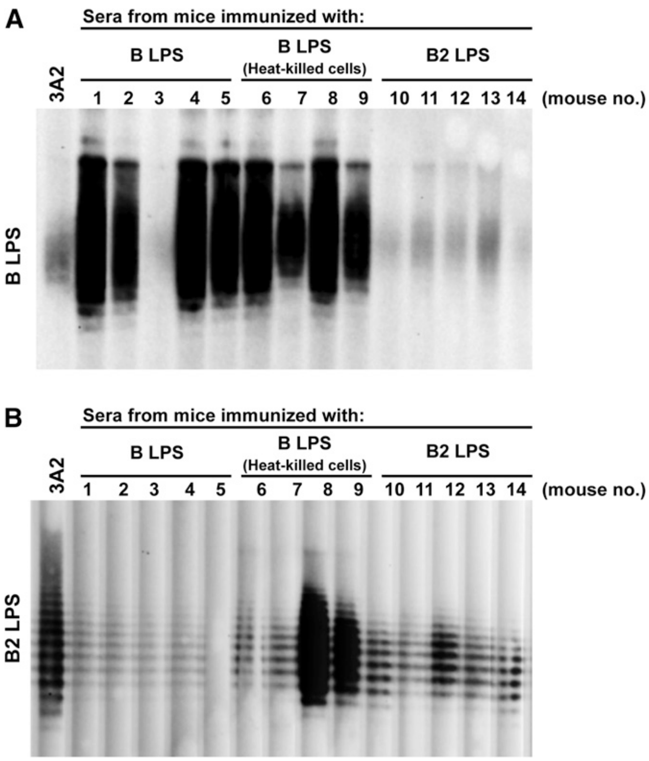

Supplemental Figure 1. Cross-reactivity of B and B2 lipopolysaccharide (LPS)–immunized mice sera. Sodium dodecyl sulfate polyacrylamide gel electrophoresis (SDS-PAGE) gels were loaded with type B (**Panel A**), and type B2 (**Panel B**) LPS. After blotting, the membranes were probed with sera from mice immunized with B LPS (lanes 1–5), heat-killed *Burkholderia ubonensis* MSMB75 (strain producing LPS type B, lanes 6–9), and B2 LPS (lanes 10–14) using Miniblotter. Monoclonal antibody (mAb) 3A2 was used as a positive control. Cross-reactivity between B and B2 LPS were observed in almost all the sera tested.

Supplemental Table 1

List of bacterial strains used in this study, their LPS phenotypes (if available), and the antigen capture immunoassay results

Bacterial species/strains	Other IDs	Source	LPS types (if known)	Typical strains detection	Atypical strians detection
Non-*Burkholderia* spp.					
Pseudomonas aeruginosa		Clinical		–	–
Acinetobacter baumannii	UF001	Clinical		–	–
A. baumannii	UF002	Clinical		–	–
A. baumannii	UF003	Clinical		–	–
A. baumannii	UF004	Clinical		–	–
A. baumannii	UF005	Clinical		–	–
A. baumannii	UF006	Clinical		–	–
A. baumannii	UF007	Clinical		–	–
A. baumannii	UF008	Clinical		–	–
A. baumannii	UF009	Clinical		–	–
A. baumannii	UF010	Clinical		–	–
A. baumannii	UF011	Clinical		–	–
A. baumannii	UF012	Clinical		–	–
A. baumannii	UF013	Clinical		–	–
A. baumannii	UF014	Clinical		–	–
A. baumannii	UF015	Clinical		–	–
Burkholderia mallei					
ATCC23344	China 7	Clinical	A (Heiss and others, 2013)	+	–
Burkholderia thailandensis					
E264		Environmental	A (Brett and others, 1998)	–	–
E555		Environmental	A (Sim and others, 2010)	+	–
MSMB121			B2 (Stone and others, 2012)	–	–
Other *Burkholderia* spp.					
Burkholderia oklahomensis C6786		Clinical		–	–
Burkholderia vietnamensis G4		Unknown		–	–
B. vietnamensis H4102		Unknown		–	–
Burkholderia pseudomallei					
1026b		Clinical	A	+	–
Bp82		1026b derivative	A (Propst and others, 2010)	+	–
5598a		Clinical		+	–
316a		Clinical		+	–
365a		Clinical		+	–
402a		Clinical		+	–
405a		Clinical		+	–
533a		Clinical		+	–
577a		Clinical		+	–
858ai		Clinical		+	–
942a		Clinical		+	–
956a		Clinical		+	–
975a		Clinical		+	–
979a		Clinical		+	–
984a		Clinical		+	–
995a		Clinical		+	–
1005a		Clinical		+	–
2085a		Clinical		+	–
2374a		Clinical		+	–
2381a		Clinical		+	–
2690a		Clinical		+	–
3013a		Clinical		+	–
3964b		Clinical		+	–
4226a		Clinical		+	–
4609a		Clinical		+	–
576a		Clinical		–	+
406e		Clinical		+	–
Bp 0004	MSHR346	Clinical		+	–
Bp 0085	DL02	Clinical		+	–
Bp 0091	DL25	Clinical		+	–
Bp 0094	DL28	Clinical		+	–
Bp 0102	DL35	Clinical		+	–
Bp 0103	DL36	Clinical		+	–
Bp 0204	RF4-Bp39	Environmental		+	–
Bp 0336	RF6-Bp15	Environmental		+	–
Bp 0412	RF23-Bp31	Environmental		+	–
Bp 0419	RF23-Bp38	Environmental		+	–
Bp 0537	RF43-Bp22	Environmental		–	+
Bp 0922	RF67-Bp1	Environmental		+	–
Bp 1270	RF85-Bp37	Environmental		+	–
Bp 2235	Int4-Bp18	Environmental		+	–

(continued)

Supplemental Table 1

Continued

Bacterial species/strains	Other IDs	Source	LPS types (if known)	Typical strains detection	Atypical strians detection
MSHR730	Bp 3994	Clinical		+	−
MSHR487	Bp 3995	Clinical	A (Tuanyok and others, 2012)	+	−
MSHR503	Bp 4001	Environmental		+	−
MSHR296	Bp 4002	Environmental	B (Tuanyok and others, 2012)	−	+
MSHR840	Bp 4003	Clinical	B2 (Tuanyok and others, 2012)	−	+
NAU14-B6	Bp 4042	Environmental		+	−
NAU21B9	Bp 4075	Environmental		+	−
NAU33A4	Bp 4099	Environmental		−	−
NAU44A6	Bp 4122	Environmental		+	−
MSHR1048	Bp 4161	Clinical		+	−
MSHR1218	Bp 4162	Clinical	A (Tuanyok and others, 2012)	+	−
MSHR1290	Bp 4164	Clinical	A (Tuanyok and others, 2012)	−	−
MSHR2408	Bp 4170	Clinical		−	−
MSHR3042	Bp 4171	Clinical		−	−
Bp 1829	INT2-Bp92	Environmental		+	−
Bp 1839	INT2-Bp129	Environmental		+	−
Bp 1842	INT2-Bp132	Environmental		+	−
Bp 1849	INT2-Bp139	Environmental		+	−
Bp 1843	INT2-Bp106	Environmental		+	−
Bp 1749	INT2-Bp39	Environmental		+	−
Bp 1870	INT2-Bp133	Environmental		+	−
Bp 1860	INT2-Bp123	Environmental		+	−
Bp 1845	INT2-Bp135	Environmental		+	−
Bp 1186	RF80-Bp1	Unknown		+	−
INT2-Bp61		Environmental		+	−
INT2-Bp127		Environmental		+	−
INT2-Bp87		Environmental		+	−
INT2-Bp19		Environmental		+	−
INT2-Bp89		Environmental		+	−
INT2-Bp214		Environmental		+	−
INT2-Bp91		Environmental		+	−
INT2-Bp190		Environmental		+	−
INT2-Bp100		Environmental		+	−
INT2-Bp24		Environmental		+	−
INT2-Bp235		Environmental		+	−
INT2-Bp217		Environmental		+	−
INT2-Bp241		Environmental		+	−
INT2-Bp223		Environmental		+	−
INT2-Bp264		Environmental		+	−
INT2-Bp38		Environmental		+	−
INT2-Bp270		Environmental		+	−
INT2-Bp48		Environmental		+	−
INT2-Bp109		Environmental		+	−
Bp 6337	NCTC13178	Clinical		+	−
Bp 6338	NCTC13179	Clinical		−	+
1710b		Clinical		+	−
1106a		Clinical		+	−
1106b		Clinical		+	−
E371	Bp354	Environmental		+	−
E645	Bp355	Environmental		+	−
E411	Bp356	Environmental		+	−
MSHR139		Clinical	B2 (Tuanyok and others, 2012)	−	+
MSHR491	Bp357	Clinical		+	−
MSHR446		Clinical	B2 (Tuanyok and others, 2012)	−	+
MSHR454		Animal	B2 (Tuanyok and others, 2012)	−	+
MSHR435	Bp358	Clinical	Rough type (Tuanyok and others, 2012)	−	−
MSHR668	Bp359	Clinical		+	−
MSHR465a	Bp360	Clinical	A (Tuanyok and others, 2012)	+	−
1468(a)	Bp361	Clinical		+	−
5041(a)	Bp363	Clinical		+	−
5242(a)	Bp364	Clinical		+	−
2259(a)	Bp367	Clinical		+	−
2517(a)	Bp368	Clinical		+	−
2703(a)	Bp369	Clinical		+	−
2411(a)	Bp370	Clinical		+	−
2444(a)	Bp371	Clinical		+	−
2431(a)	Bp372	Clinical		+	−
1641(a)	Bp373	Clinical		+	−
1130(a)	Bp374	Clinical		+	−

(continued)

Supplemental Table 1
Continued

Bacterial species/strains	Other IDs	Source	LPS types (if known)	Typical strains detection	Atypical strians detection
415a	Bp375	Clinical		+	–
699c	Bp377	Clinical		+	–
699d	Bp378	Clinical		+	–
1142a	Bp379	Clinical		+	–
1142b	Bp380	Clinical		+	–
2613a	Bp84	Clinical	A (Tuanyok and others, 2012)	+	–
2614a	Bp85	Clinical	A (Tuanyok and others, 2012)	+	–
2617a	Bp86	Clinical	A (Tuanyok and others, 2012)	+	–
2618a	Bp87	Clinical	A (Tuanyok and others, 2012)	+	–
2625a	Bp88	Clinical	A (Tuanyok and others, 2012)	+	–
2637a	Bp89	Clinical		+	–
2640a	Bp90	Clinical		+	–
2650a	Bp91	Clinical	A (Tuanyok and others, 2012)	+	–
2660a	Bp92	Clinical	A (Tuanyok and others, 2012)	+	–
2661a	Bp93	Clinical	A (Tuanyok and others, 2012)	+	–
2665a	Bp94	Clinical	A (Tuanyok and others, 2012)	+	–
2667a	Bp95	Clinical	A (Tuanyok and others, 2012)	+	–
2668a	Bp96	Clinical	A (Tuanyok and others, 2012)	+	–
2670a	Bp97	Clinical	A (Tuanyok and others, 2012)	+	–
2671a	Bp98	Clinical	A (Tuanyok and others, 2012)	+	–
2673a	Bp99	Clinical	A (Tuanyok and others, 2012)	+	–
2674a	Bp100	Clinical	A (Tuanyok and others, 2012)	+	–
2682a	Bp 102	Clinical	A (Tuanyok and others, 2012)	+	–
2685a	Bp 103	Clinical	A (Tuanyok and others, 2012)	+	–
2689b	Bp 104	Clinical	A (Tuanyok and others, 2012)	+	–
2692a	Bp 105	Clinical	A (Tuanyok and others, 2012)	+	–
2694a	Bp 106	Clinical	A (Tuanyok and others, 2012)	+	–
2698a	Bp 107	Clinical	A (Tuanyok and others, 2012)	+	–
2708a	Bp 109	Clinical	A (Tuanyok and others, 2012)	+	–
2717a	Bp 110	Clinical	A (Tuanyok and others, 2012)	+	–
2719a	Bp 111	Clinical	A (Tuanyok and others, 2012)	+	–
2764b	Bp 112	Clinical		+	–
2769a	Bp 113	Clinical	A (Tuanyok and others, 2012)	+	–
E0008	Bp 114	Environmental	A (Tuanyok and others, 2012)	+	–
E0016	Bp 115	Environmental	A (Tuanyok and others, 2012)	+	–
E0021	Bp 116	Environmental	A (Tuanyok and others, 2012)	+	–
E0024	Bp 117	Environmental	A (Tuanyok and others, 2012)	+	–
E0031	Bp 118	Environmental	A (Tuanyok and others, 2012)	+	–
E0034	Bp 119	Environmental	A (Tuanyok and others, 2012)	+	–
E0037	Bp 120	Environmental	A (Tuanyok and others, 2012)	+	–
E0181	Bp 121	Environmental	A (Tuanyok and others, 2012)	+	–
E0183	Bp 122	Environmental	A (Tuanyok and others, 2012)	+	–
E0237	Bp 124	Environmental	A (Tuanyok and others, 2012)	+	–
E0241	Bp 125	Environmental	A (Tuanyok and others, 2012)	+	–
E0279	Bp 126	Environmental	A (Tuanyok and others, 2012)	+	–
E0342	Bp 127	Environmental	A (Tuanyok and others, 2012)	+	–
E0345	Bp 128	Environmental	A (Tuanyok and others, 2012)	+	–
E0350	Bp 129	Environmental		+	–
E0356	Bp 130	Environmental	A (Tuanyok and others, 2012)	+	–
E0366	Bp 131	Environmental	A (Tuanyok and others, 2012)	+	–
E0371	Bp 132	Environmental	A (Tuanyok and others, 2012)	+	–
E0372	Bp 133	Environmental	A (Tuanyok and others, 2012)	+	–
E0377	Bp 134	Environmental	A (Tuanyok and others, 2012)	+	–
E0378	Bp 135	Environmental	A (Tuanyok and others, 2012)	+	–
E0380	Bp 136	Environmental		+	–
E0383	Bp 137	Environmental	A (Tuanyok and others, 2012)	+	–
E0384	Bp 138	Environmental	A (Tuanyok and others, 2012)	+	–
E0386	Bp 139	Environmental		+	–
E0393	Bp 140	Environmental	A (Tuanyok and others, 2012)	+	–
E0394	Bp 141	Environmental	A (Tuanyok and others, 2012)	+	–
E0396	Bp 142	Environmental	A (Tuanyok and others, 2012)	+	–
E0411	Bp 143	Environmental	A (Tuanyok and others, 2012)	+	–
PHLS 83	Bp856	Clinical		+	–
Bp21651	Bp857	Clinical		–	–

(continued)

Supplemental Table 1

Continued

Bacterial species/strains	Other IDs	Source	LPS types (if known)	Typical strains detection	Atypical strians detection
Negative control					
No sample				–	–
No sample				–	–
No sample				–	–
No sample				–	–
No sample				–	–
No sample				–	–
No sample				–	–
No sample				–	–
No sample				–	–
No sample				–	–

LPS = lipopolysaccharide.
